# Leptin Signaling Is Required for Adaptive Changes in Food Intake, but Not Energy Expenditure, in Response to Different Thermal Conditions

**DOI:** 10.1371/journal.pone.0119391

**Published:** 2015-03-10

**Authors:** Karl J. Kaiyala, Kayoko Ogimoto, Jarrell T. Nelson, Michael W. Schwartz, Gregory J. Morton

**Affiliations:** 1 Department of Oral Health Sciences, School of Dentistry, University of Washington, Seattle, WA, United States of America; 2 Diabetes and Obesity Center of Excellence, Department of Medicine, University of Washington, Seattle, WA, United States of America; Hosptial Infantil Universitario Niño Jesús, CIBEROBN, SPAIN

## Abstract

Survival of free-living animals depends on the ability to maintain core body temperature in the face of rapid and dramatic changes in their thermal environment. If food intake is not adjusted to meet the changing energy demands associated with changes of ambient temperature, a serious challenge to body energy stores can occur. To more fully understand the coupling of thermoregulation to energy homeostasis in normal animals and to investigate the role of the adipose hormone leptin to this process, comprehensive measures of energy homeostasis and core temperature were obtained in leptin-deficient *ob/ob* mice and their wild-type (WT) littermate controls when housed under cool (14°C), usual (22°C) or ∼ thermoneutral (30°C) conditions. Our findings extend previous evidence that WT mice robustly defend normothermia in response to either a lowering (14°C) or an increase (30°C) of ambient temperature without changes in body weight or body composition. In contrast, leptin-deficient, *ob/ob* mice fail to defend normothermia at ambient temperatures lower than thermoneutrality and exhibit marked losses of both body fat and lean mass when exposed to cooler environments (14°C). Our findings further demonstrate a strong inverse relationship between ambient temperature and energy expenditure in WT mice, a relationship that is preserved in *ob/ob* mice. However, thermal conductance analysis indicates defective heat retention in *ob/ob* mice, irrespective of temperature. While a negative relationship between ambient temperature and energy intake also exists in WT mice, this relationship is disrupted in *ob/ob* mice. Thus, to meet the thermoregulatory demands of different ambient temperatures, leptin signaling is required for adaptive changes in both energy intake and thermal conductance. A better understanding of the mechanisms coupling thermoregulation to energy homeostasis may lead to the development of new approaches for the treatment of obesity.

## Introduction

Although the systems governing thermoregulation and energy homeostasis are anatomically and functionally distinct, highly coordinated interactions between them are required if body energy reserves are to be maintained in the face of changing thermal environments. This coordination between thermoregulatory and energy homeostasis systems is especially critical for small endothermic mammals (such as mice) owing to their combination of high mass-specific metabolic rates and large surface area to volume ratios. During cold exposure, for example, the energy needs associated with defense of core body temperature increase substantially [1] and, should energy intake fail to increase sufficiently to offset the increase of energy expenditure, the resultant depletion of body fuel stored as adipose tissue will be rapid and potentially life threatening. Conversely, warm environments will cause excessive weight gain unless the declining need for heat production is offset by appropriately reduced food intake.

Because the adiposity hormone leptin participates in the regulation of both energy homeostasis [2] and thermoregulation [3], leptin-deficient *ob/ob* mice are characterized not only by severe obesity, but by hypothermia when housed at temperatures below thermoneutrality. Leptin is therefore an attractive potential mediator of the coupling between thermoregulatory and energy homeostasis systems that ensures stability of both core temperature and body fat stores across a wide range of environmental temperatures. In the current work, we sought to quantitatively and comprehensively compare the response of the thermoregulatory and energy homeostasis systems between leptin-deficient *ob/ob* mice and their wild-type (WT) littermate controls when subjected to housing in each of three thermal environments: at room temperature (22°C), at ∼thermoneutrality (30°C), and at a cool temperature (14°C).

Our findings demonstrate that whereas body mass, body composition and body temperature are held more or less constant in control mice irrespective of ambient temperature, *ob/ob* mice exhibit marked swings of energy balance, body mass and core temperature when confronted with different thermal environments. Consistent with earlier work [4,5], *ob/ob* mice became hypothermic when housed at temperatures below thermoneutrality, but unexpectedly, energy expenditure was regulated similarly in *ob/ob* and control mice across the different thermal environments. Our data also suggest that the failure of *ob/ob* mice to maintain core temperature despite increasing energy expenditure appropriately involves an increase of whole body thermal conductance relative to WT mice, implicating a role for leptin in heat conservation effectors. Moreover, *ob/ob* mice displayed a profound inability to adjust food intake to meet the changing energy needs associated with housing across a range of temperatures. Consequently, these animals lost disproportionate amounts of both lean and fat mass when housed in the cold, whereas they gained weight excessively when housed at ∼thermoneutrality. These observations identify a critical role for leptin not only in the maintenance of core body temperature, but also in the ability to dynamically alter food intake in response to changing thermal environments. By comparison, the ability of mice to adjust energy expenditure, but not thermal conductance, in response to changing thermal needs appears to be mediated via a leptin-independent mechanism.

## Materials and Methods

### Animals

Studies were conducted using adult, male C57/Bl6 and leptin-deficient *ob/ob* mice on the C57/Bl6 background strain (Jackson Laboratories, Bar Harbor, ME). All animals were housed individually under specific pathogen-free conditions in a temperature-controlled room with a 12:12h light:dark cycle and provided ad libitum (AL) access to standard laboratory chow (PMI Nutrition International, MO) and water unless otherwise stated.

### Ethics information

All procedures were performed in accordance with NIH Guidelines for the Care and Use of Animals and were approved by the Animal Care Committee at the University of Washington.

### Body composition analysis

Measures of body lean and fat mass were determined in live, conscious animals using quantitative magnetic resonance spectroscopy (QMR) (EchoMRI 3-in-1; Echo MRI, TX) made available through the University of Washington Nutrition Obesity Research Center (NORC) Energy Balance and Glucose Metabolism (EBGM) Core [6]. QMR measures of fat content were validated each measurement day by scanning a calibration holder containing a known amount of fat.

### Indirect calorimetry

Separate cohorts of *ob/ob* mice and littermate controls were acclimated to metabolic cages prior to measurement of energy expenditure using a computer controlled indirect calorimetry system (Promethion, Sable Systems, Las Vegas, NV) located in the EBGM Core of the NORC at the University of Washington as described in detail previously [7–9]. Calorimeter cages (similar to home cages with bedding) were each equipped with water bottles and food hoppers connected to load cells for continuous food and water intake monitoring and housed in a temperature- and humidity-controlled cabinet (Caron Products and Services, Marietta, OH) [7–9]. O_2_ consumption and CO_2_ production were measured for each animal for 1 min at 10-min intervals as previously described [7–9]. Respiratory quotient (RQ) was calculated as the ratio of CO_2_ production to O_2_ consumption. Energy expenditure was calculated using the Weir equation [10]. Energy expenditure was not adjusted to correct for differences of body size, as there was no overlap in body or fat mass between groups, and statistical comparisons were based on the response to different thermal environments within each genotype. This decision was made in part because statistical adjustment of energy expenditure for lean mass within groups exhibiting similar mean values for this covariate would not fully eliminate confounding by the large differences in total mass or fat mass between groups [11,12]. Ambulatory activity was determined continuously, with consecutive adjacent infrared beam breaks in the x-, y- and z-axes scored as an activity count that was recorded every 10 min as previously described [7–9]. Data acquisition and instrument control were coordinated by MetaScreen v.2.0.0.9 and raw data was processed using ExpeData v.1.6.4 (Sable Systems) using an analysis script documenting all aspects of data transformation. Photoperiod-averaged 24-h data were calculated from 6 data points averaged over a 1-h period, and these in turn were averaged over the 3 consecutive days for each ambient temperature.

### Core temperature

To quantify the effect of ambient temperature on core temperature, adult male *ob/ob* mice and their littermate controls underwent implantation of body temperature transponders (Starr Life Science Corp, Oakmont, PA) in the peritoneal cavity. Animals were allowed at least 1 wk to recover and were subsequently acclimated to calorimeter cages housed in temperature- and humidity-controlled cabinets (Caron Products and Services, Marietta, OH) prior to study. Transponder signals encoding body temperature were sensed by a receiver positioned underneath each cage and analyzed using VitalView software. Photoperiod-averaged core temperature was computed as the mean ± SEM of all values obtained throughout each photoperiod at each ambient temperature. Energy expenditure data was recorded as described above.

### Thermal conductance

To provide an index of the ease with which heat flows from the body core to the environment, we calculated whole body thermal conductance. While we did not measure heat loss, conductance can be calculated based on energy expenditure whenever the mean energy expenditure rate equals the mean heat loss rate as occurs when the means encompass time intervals that involve very little or no net change in core temperature (e.g., 24 h periods). We quantified conductance using two methods: *1)* the simple traditional method whereby each animal’s conductance is calculated as energy expenditure divided by its core temperature minus ambient temperature difference [13], and *2)* using a regression approach wherein energy expenditure and conductance were modeled in terms of non-linear power (allometric) functions of core-minus ambient-temperature difference, a choice motivated by the ubiquity of allometric scaling of biophysical processes [14,15]. Additional details are available in S1 Fig.

### Experimental protocols

**Fig 1:**
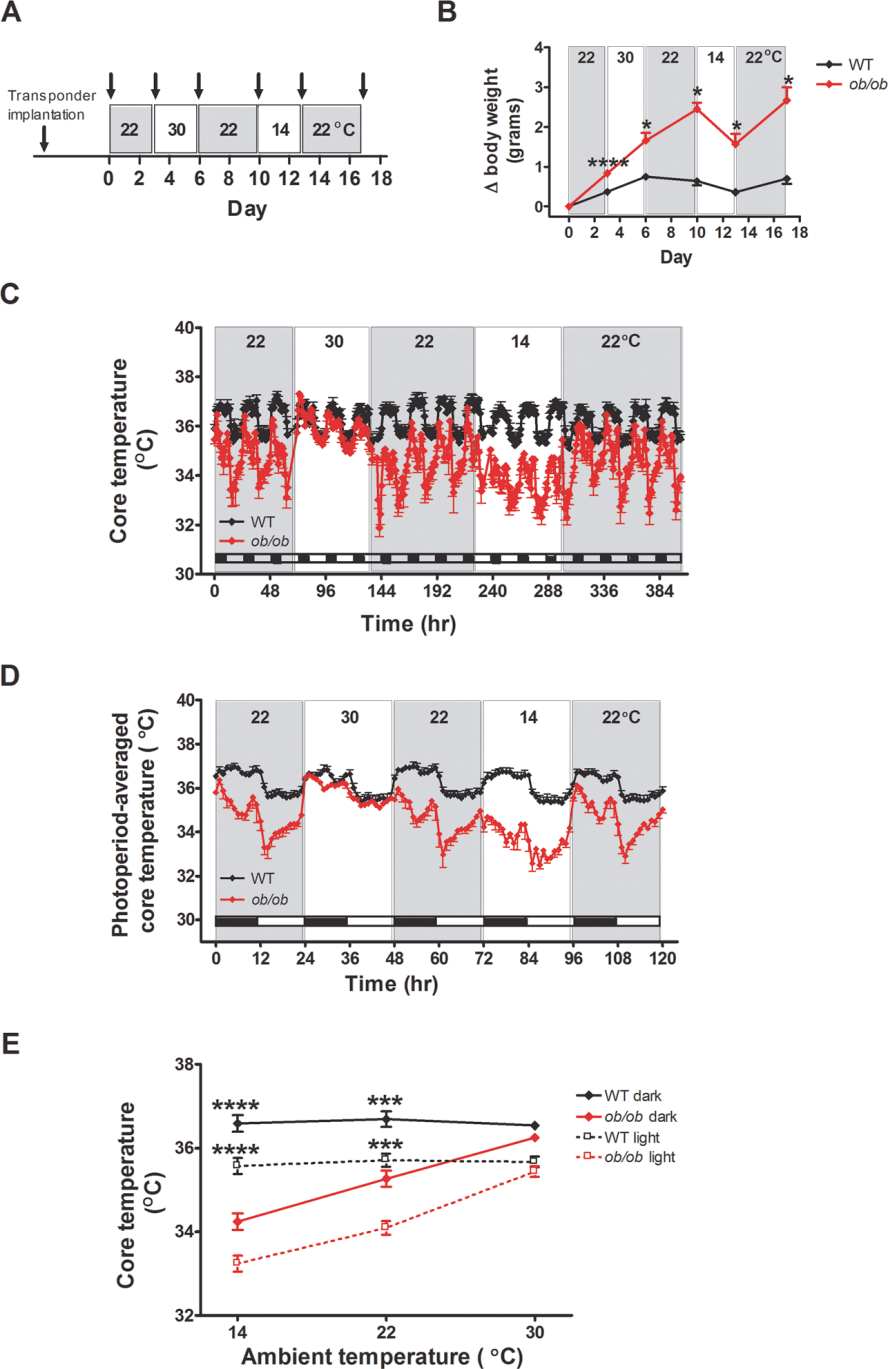
Leptin deficient animals fail to defend normothermia at temperatures lower than thermoneutrality. (**A**) Study design, (**B**) change in body weight, (**C**) core body temperature across all photoperiods, (**D**) photoperiod-averaged core body temperature profiles, and (**E**) the relationship between core temperature and ambient temperature in adult male *ob/ob* mice and wild-type (WT) littermate controls implanted with temperature transponders for continuous measures of core body temperature and housed under different ambient temperature conditions (n = 7/group). Arrowheads mark measures of body weight. Mean±SEM. ****p<0.0001, ***p<0.001, *p<0.05.

**Fig 2:**
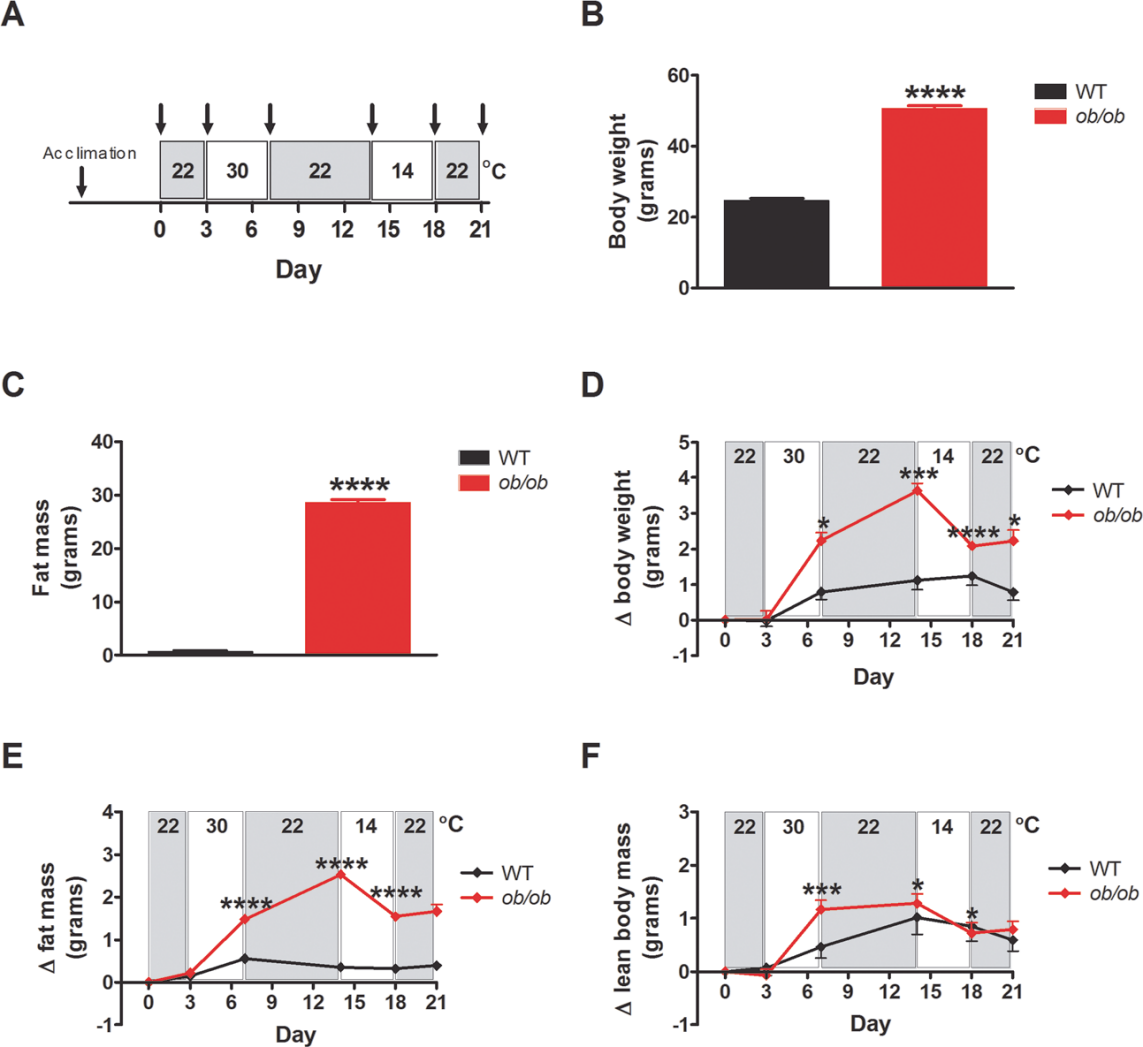
Leptin deficient animals are unable to maintain energy homeostasis in response to a thermal challenge. (**A**) Study design, (**B**) body weight, (**C**) fat mass, (**D**) change in body weight, (**E**) change in fat mass and (**F**) change in lean body mass in adult male *ob/ob* mice and wild-type (WT) littermate controls housed under different ambient temperature conditions (n = 8/group). Arrowheads mark measures of body weight and composition. Mean±SEM. ****p<0.0001, ***p<0.001, *p<0.05.

Following body temperature transponder implantation and acclimation to cages within the temperature- and humidity-controlled chambers, body temperature was recorded for 68 h with the temperature maintained at 22.0 ± 0.1°C. Subsequently, the temperature was raised over a 4-h interval to 30.0 ± 0.1°C and animals remained at this temperature for an additional 68 h. Based on thermal preference data, this temperature is, or is close to thermoneutral in WT mice [13], although this has yet to be established in *ob/ob* mice. In addition, since thermoneutral temperatures may differ between the light and dark cycle, we consider 30°C to be approximately thermoneutral for both genotypes (referred to subsequently as “∼thermoneutrality”). Subsequently, the temperature was lowered over a 4-h time interval back to 22.0 ± 0.1°C, where it remained for an additional 68 h, and then reduced again to 14.0 ± 0.1°C for 68 h before returning back to 22.0 ± 0.1°C (Fig. 1A). For measures of energy expenditure, food intake and locomotor activity, separate groups of *ob/ob* and littermate control mice (that did not undergo transponder implantation because this makes body composition analysis impossible) were acclimated to metabolic cages housed in temperature- and humidity-controlled chambers and subjected to the identical protocol of changing thermal environments (Fig. 2A).

### Statistical Analyses

Results are expressed as mean ± SEM. Significance was established as two-sided probability values of less than 0.05. For statistical comparisons involving core temperature, energy expenditure or food intake, data obtained during the three 22°C test periods were reduced into average light and dark photoperiod components for each mouse. Genotype and ambient temperature were both modeled as fixed effects. To handle the repeated measure (ambient temperature) and the error structure of the data appropriately, linear mixed model analysis with an unstructured covariance matrix was employed to evaluate the significance of genotype, ambient temperature and the genotype by ambient temperature interaction term. Regression modeling to derive the mathematical relationships between energy expenditure or thermal conductance and the core minus ambient temperature difference also made use of linear mixed model analysis but employed a compound symmetry covariance matrix. Linear mixed model analyses were undertaken using (SPSS version 22, IBM Corp., Somers, NY). Comparisons between genotypes within each ambient temperature condition were evaluated by custom contrasts. Statistical analyses of body composition data were performed using Statistica (version 7.1; StatSoft, Inc., Tulsa, OK). A one-way ANOVA with a least significant difference post hoc test was used to compare mean values between multiple groups and a two-sample unpaired Student’s t test was used for two-group comparisons.

## Results

### Effect of ambient temperature on core body temperature and body weight

As a first step to investigate the role of leptin in mechanisms coupling energy homeostasis to thermoregulation, we determined the effect on core body temperature and body mass of housing *ob/ob* mice and their littermate controls for 68h in each of 3 different thermal environments (Fig. 1A). Consistent with previous studies [16–18], wild-type (WT) littermate control mice effectively adjusted energy intake to meet the shifting energy requirements imposed by changes of environmental temperature, as evidenced by maintenance of stable body weight at each temperature. By comparison, *ob/ob* mice exhibited significantly greater body weight gain than controls both at room temperature (22°C) and at ∼thermoneutrality (30°C), whereas cold exposure (14°C) induced significant weight loss in *ob/ob* mice, but not controls (Fig. 1B).

Analysis of core temperature within each photoperiod revealed significant effects of genotype, ambient temperature and the genotype by ambient temperature interaction (genotype: F (1, 12) ≥ 43.1; p<0.0001; ambient temperature: (F (2,12) ≥ 42.6; p < 0.0001; interaction: F (2,12) ≥ 38.3; <0.0001)). WT mice defended the euthermic state (defined on the basis of mean core temperature) with remarkable robustness in the face of reduced ambient temperature whereas *ob/ob* mice exhibited hypothermia at 22°C that became marked at 14°C (p for trend < 0.0001 in both photoperiods; Fig. 1C-E). In contrast, core temperature did not differ significantly between groups in either photoperiod when housed at 30°C (Fig. 1E), although there was a trend for slightly lower core temperature in *ob/ob* vs. WT mice in the dark photoperiod (by minus 0.286 ± 0.140°C; p = 0.06).

As expected [19], WT mice not only maintained core temperature within a normal range when tested at different ambient temperatures, they also preserved a diurnal temperature rhythm characterized by a decline in core temperature beginning at light cycle onset, coincident with the transition from being awake, active and eating, to a period of sleep, fasting, reduced activity and energy conservation, before subsequently returning to baseline at the onset of the dark cycle (Fig. 1C, D) [20]. Despite exhibiting hypothermia, this diurnal core temperature rhythm was preserved in *ob/ob* mice (Fig. 1C, D). Although these findings are consistent with previous evidence of defective thermoregulation in cold-exposed *ob/ob* mice [4,5], this defect was largely rescued by housing *ob/ob* mice under ∼thermoneutral conditions (30°C), as their core temperature means were not statistically different than those of controls in either the dark or light photoperiods in this setting (Fig. 1E). Relative to wild-type mice, *ob/ob* mice also exhibited a more dramatic decline in core temperature following a decline in environmental temperature, particularly from 30°C to 22°C, than controls, and they were also subsequently unable to return to baseline temperature as controls consistently did (Fig. 1C). These findings suggest *1)* at room temperature, *ob/ob* mice are unable to engage the normal adaptive heat producing and/or heat conserving mechanisms required to support core temperature, and *2)* their body temperature increases into the normal range when housed at ∼thermoneutrality, because these adaptive responses are no longer needed. When animals are transferred back to 22°C, however, these defects again become apparent and are manifest as a greater initial decline in core temperature and a slower than normal recovery to a new baseline temperature, which is below that of controls. Future studies are warranted to identify specific leptin-responsive mechanisms that contribute to adaptive thermogenesis and the time-course over which they are engaged following a change in ambient temperature. Collectively, these observations suggest that *1)* the intrinsic core temperature target in *ob/ob* mice is the same or similar to that of WT mice, *2) ob/ob* mice fail to defend this normal value in the face of reduced ambient temperatures, and *3)* this thermoregulatory defect is associated with a compromised capacity to adjust energy balance so as to maintain body weight stability in the face of a thermal challenge.

### Effect of ambient temperature on body composition and energy balance

To more fully characterize the impact of changing thermal environments on energy balance in normal and *ob/ob* mice, we measured the effects of different ambient temperatures on body composition, energy intake and energy expenditure in both genotypes (Fig. 2A). As expected, the increase of body weight at baseline exhibited by *ob/ob* mice was due entirely to increased body fat mass without differences of lean body mass (Fig. 2B, C) and, when housed for 68 h at either 22°C or 30°C, the excess body weight gain displayed by *ob/ob* mice relative to WT controls reflected further increases of body fat stores along with increased lean body mass (Fig. 2D, E). When housed at 14°C, however, *ob/ob* mice exhibited a marked decrease of body weight due to losses of both fat and lean mass (Fig. 2D-F), whereas control mice maintained both fat and lean mass within a very narrow range. These findings demonstrate that *ob/ob* mice are unable to maintain energy homeostasis in response to a thermal challenge, with a tendency to increase body fat mass when housed in warm temperatures and to lose excessive amounts of both lean and fat mass during cold exposure.

### Effect of ambient temperature on energy intake

**Fig 3:**
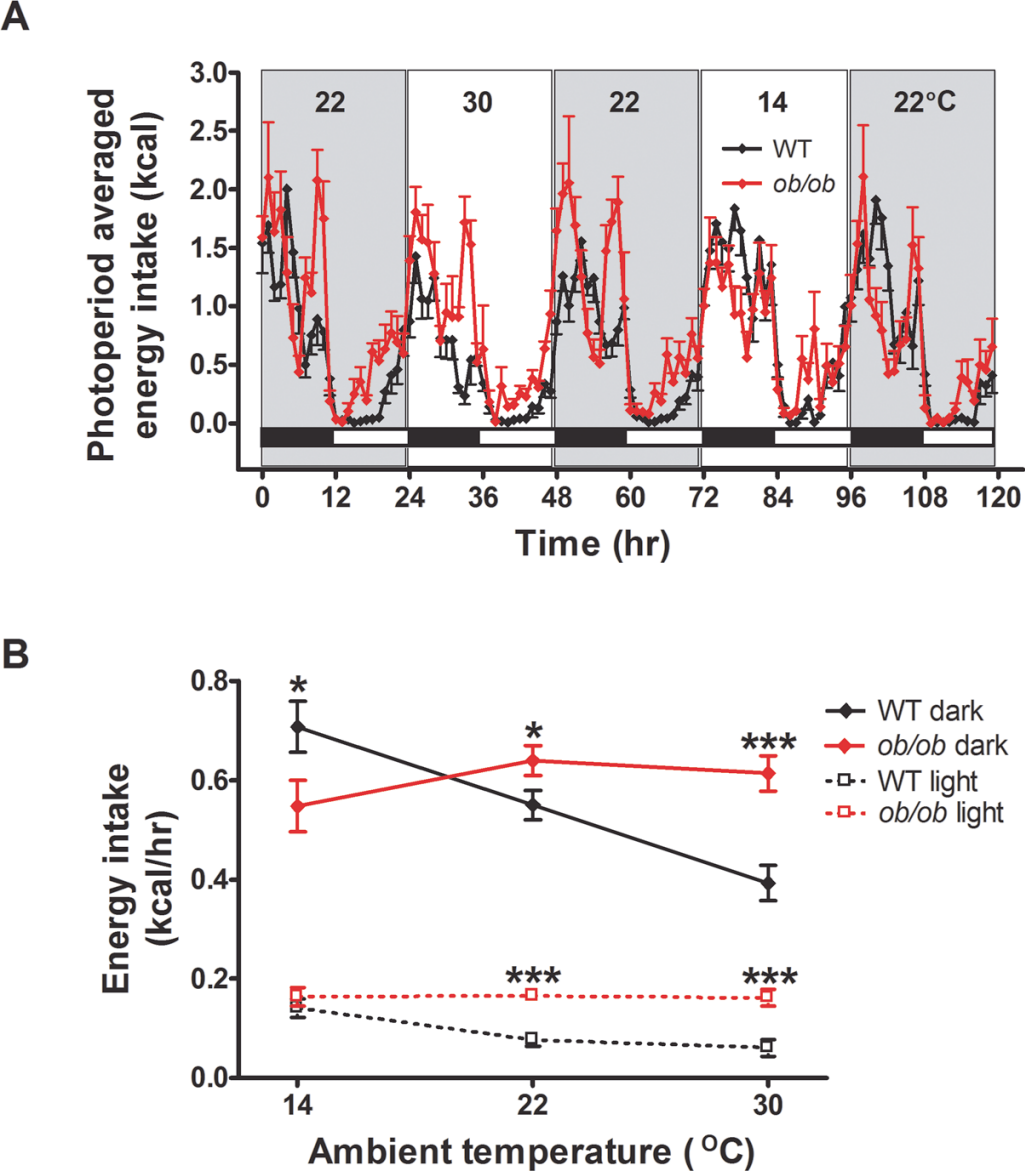
Leptin signaling is required for adaptive changes in energy intake in response to a thermal challenge. (**A**) Photoperiod-averaged energy intake profiles and (**B**) the relationship between energy intake and ambient temperature in adult male *ob/ob* mice and wild-type (WT) littermate controls housed under different ambient temperature conditions (n = 8/group). Mean±SEM. ***p<0.001, *p<0.05.

To better characterize the energy balance defect induced by leptin deficiency in differing thermal environments, we measured both energy expenditure (by indirect calorimetry) and energy intake continuously during exposure to each of the three temperatures. Illustrating the robust coupling of energy homeostasis to temperature homeostasis in normal (leptin-intact) mice, dark cycle food intake increased linearly and sharply with decreasing ambient temperature in WT mice (p for trend<0.0001), achieving values ∼2-fold greater at 14°C than 30°C (Fig. 3). It is also worth noting that, consistent with previous findings [7,18], WT mice exhibited a compensatory decrease of mean food intake when the environmental temperature was increased from 22°C to 30°C, an effect that was evident during both dark and light cycles (by ∼24% and 35%, respectively; Fig. 3A, B). These findings underscore the concept that in normal mice housed at normal laboratory temperature, a substantial proportion of their food intake and energy expenditure is devoted to thermoregulation [7]. Further, the observation that these compensatory food intake adjustments became apparent during the first dark cycle after the ambient temperature was raised or lowered implies the existence of as yet unknown mechanisms for rapid matching of energy intake to meet thermoregulatory requirements (as stressed recently [7]) that are operative throughout the exposure to altered temperature.

In contrast to the robust increase observed in WT controls, energy intake in *ob/ob* mice actually tended to decrease with cold stress, but overall changed little across the range of ambient temperatures (Fig. 3A, B; F(2,14) = 0.88; p = 0.44). Consequently, *ob/ob* mice rapidly lose body mass in the cold, while accumulating excess body fat mass in a thermoneutral environment. Congenital leptin deficiency, therefore, impairs the ability to adjust energy intake to meet the changing heat production requirements imposed by variation in ambient temperature.

### Effect of ambient temperature on energy expenditure

**Fig 4:**
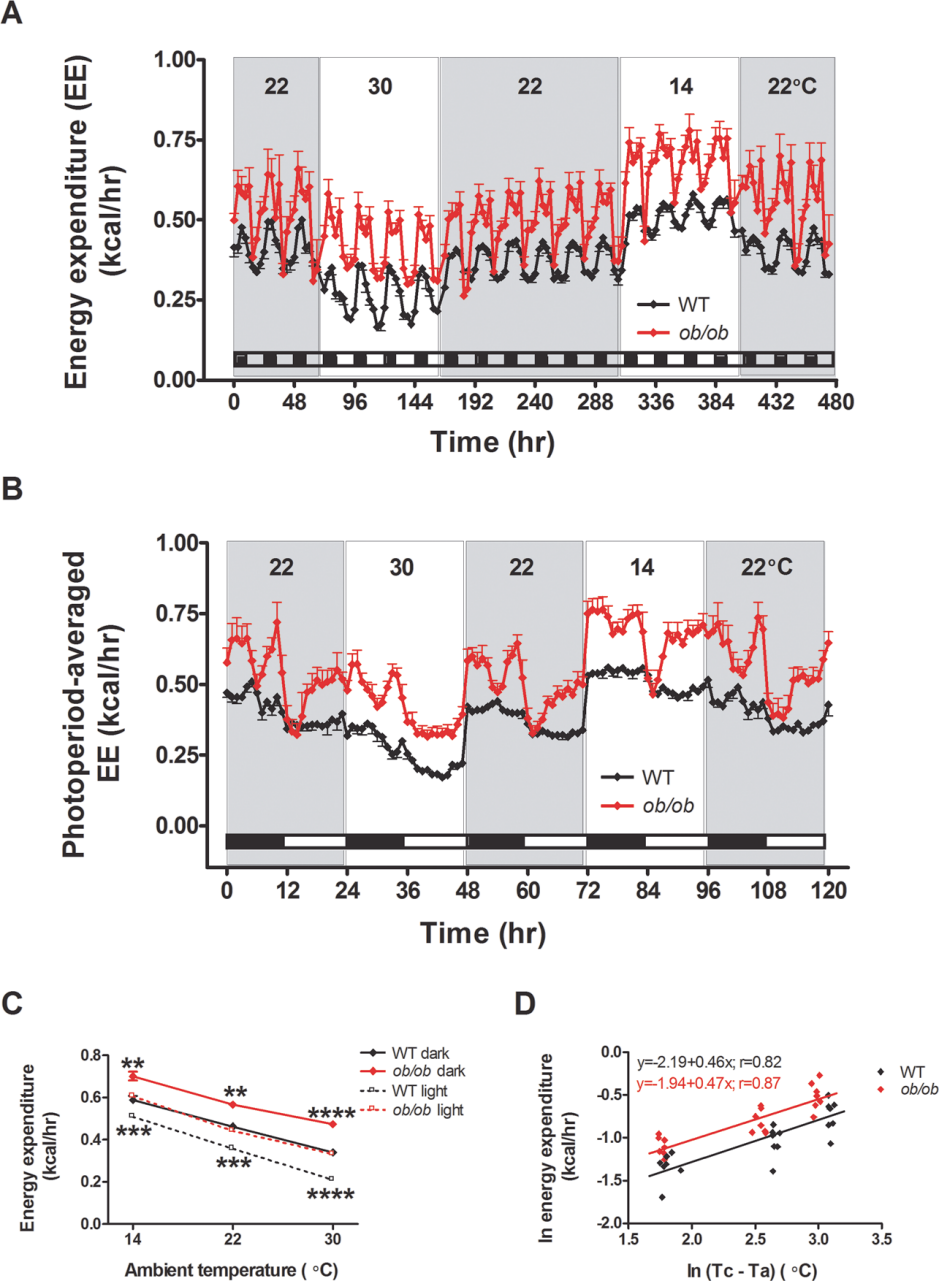
Leptin deficiency does not impair the whole body heat production response to cold stress in mice. (**A**) Energy expenditure across all photoperiods, (**B**) photoperiod-averaged energy expenditure (EE) profiles and (**C**) the relationship between energy expenditure and ambient temperature in adult male *ob/ob* mice and wild-type (WT) littermate controls housed under different ambient temperature conditions (n = 8/group). (D) Regression of natural log (ln) of mean 24h EE values on the natural log of the mean difference between core temperature (Tc) and ambient temperature (Ta) (see S1 Fig.). Mean±SEM. ****p<0.0001, ***p<0.001, **p<0.01.

Unexpectedly, this defect in the response of *ob/ob* mice to different thermal environments did not extend to the control of energy expenditure. Analysis of energy expenditure within each photoperiod revealed significant effects of genotype, ambient temperature, and the genotype by ambient temperature interaction (genotype: F (1, 14) ≥ 23.6; p≤0.0003; ambient temperature: (F (2,14) ≥ 727.7; p<0.0001; interaction: F (2,14) ≥ 9.1; p≤0.003)). Although the genotype by ambient temperature interaction was significant in both photoperiods when ambient temperature was treated as a factor, the effect of lowered ambient temperature to increase energy expenditure was nonetheless quantitatively similar in *ob/ob* and WT groups (Fig. 4A-C). Indeed, the energy expenditure increase in response to reduced ambient temperature was remarkably similar between genotypes (Fig. 4C). When ambient temperature was modeled as a covariate rather than as a factor, the interaction term was non-significant for the dark photoperiod analysis (p = 0.38) but remained significant for the light photoperiod (p = 0.002; however the difference between slopes of energy expenditure on ambient temperature was small: 0.186 kcal/h (WT) vs. 0.156 kcal/h (*ob/ob*) per 10°C decrease in ambient temperature).

Based on theoretical and empirical grounds, the core minus ambient temperature difference is a key determinant of heat loss (and therefore of metabolic rate) in mice and other species [21]. Our analysis revealed that energy expenditure in both *ob/ob* and WT mice are parallel linear functions of the core minus ambient temperature difference when considered on logarithmic scales (Fig. 4D), meaning that these variables conform to non-linear power (allometric) functions on the usual number scale. The allometric equations for each genotype are presented in S1 Fig. To our knowledge, this is the first demonstration that energy expenditure scales as an allometric function of the core minus ambient temperature difference. Previous analyses have focused on energy expenditure as linear functions of ambient temperature or as allometric functions of body mass [21,22]. Our analysis reveals that for any particular value of the difference between core and ambient temperature, both energy expenditure and the increase of energy expenditure per unit increase in the temperature difference are 31.8% higher in *ob/ob* mice than in controls.

The finding that energy expenditure was ∼32% greater in *ob/ob* compared to WT mice irrespective of the temperature difference and yet *ob/ob* mice maintain subnormal core temperature when housed at sub-thermoneutral ambient temperatures strongly suggests that leptin deficiency increases thermal conductance. In addition, components of the elevated energy expenditure in *ob/ob* mice likely reflect the fact that they have greater tissue mass to keep warm compared to WT mice (∼93% in our analysis) and that their reduced core temperature in sub-thermoneutral environments represents an “error signal” that stimulates energy expenditure. Additional studies are therefore needed to delineate mechanisms underlying the impact of leptin on the relationship between energy expenditure and core body temperature.

**Fig 5:**
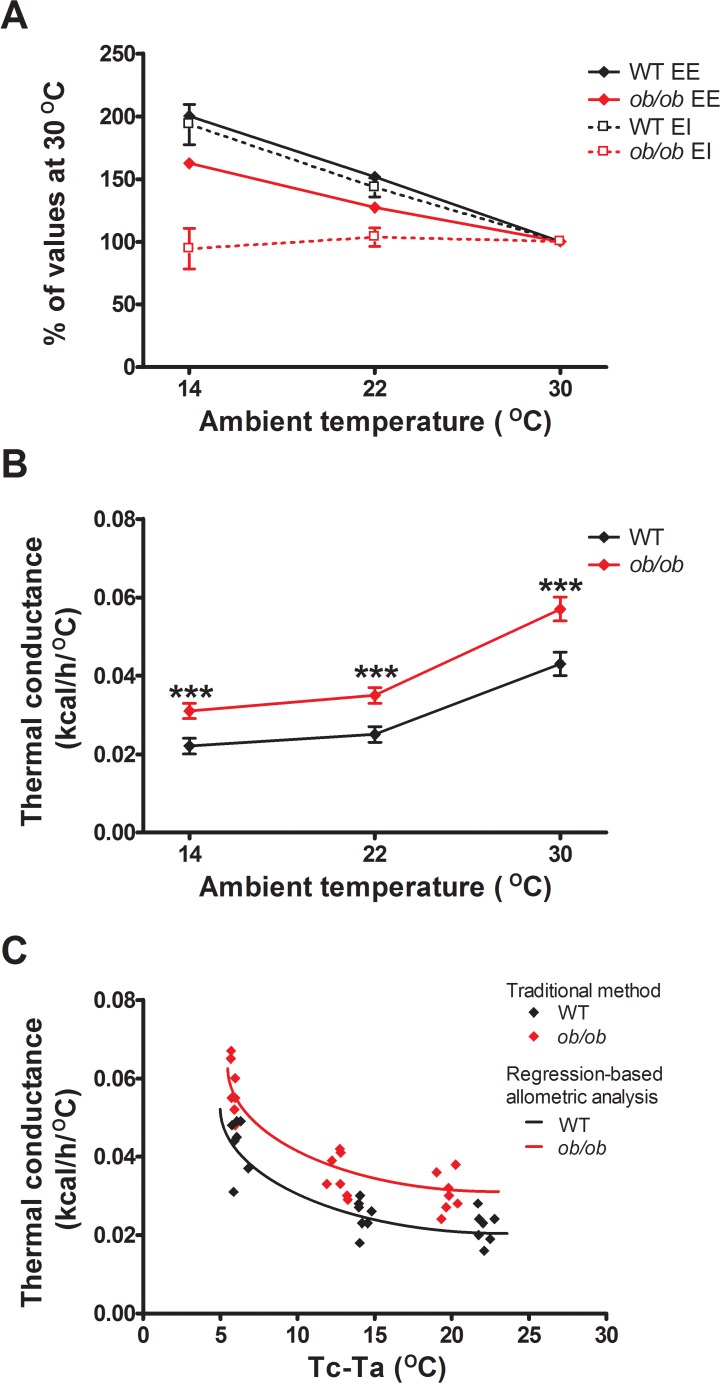
Adaptive changes of energy intake, but not energy expenditure in response to different ambient temperatures, requires leptin signaling. (A) Energy expenditure (EE) and energy intake (EI) expressed as percentages of the values observed at thermoneutrality vs. ambient temperature and (B) whole body thermal conductance calculated from mean 24h energy expenditure *vs*. ambient temperature in wild-type (WT) and leptin-deficient *ob/ob* mice using the traditional method [13] (n = 8/group). (C) Comparison of traditional *vs*. regression methods for estimating whole body thermal conductance as functions of the core minus ambient (Tc—Ta) temperature difference based on 24h data (see S1 Fig.). Mean±SEM. ***p<0.001.

Our finding that the response of energy expenditure to a change of ambient does not appear to require leptin signaling warrants additional comment. Thus, when the ambient temperature is raised from 22°C to ∼thermoneutrality, the reduction of energy expenditure is similar in WT and *ob/ob* mice. Moreover, similar to WT mice, *ob/ob* mice also exhibit an unexpectedly intact energy expenditure response to cold exposure. These findings suggest that unlike their inability to appropriately adjust food intake, *ob/ob* mice effectively and appropriately adjust energy expenditure in response to changes of ambient temperature (Fig. 5A).

The finding that *ob/ob* mice become hypothermic despite increasing energy expenditure appropriately implies that they are unable to conserve heat normally when housed at temperatures below thermoneutrality. To test this hypothesis, we calculated whole body thermal conductance in both genotypes using both a traditional method and a regression-based approach. Defined as the ease with which heat is lost to the environment, thermal conductance determines the metabolic heat production requirement for an endotherm to remain normothermic in any given thermal environment [13]. As predicted, the traditional method revealed that *ob/ob* mice exhibit higher whole body thermal conductance (p<0.0001) across each of the 3 temperatures (Fig. 5B). The allometric analysis of conductance (K) (Fig. 5C and S1 Fig.) revealed that conductance in *ob/ob* mice was ∼32% higher than in WT controls (p = 0.004). Together, these findings suggest that leptin deficiency has only a significant effect on the relationship between the driving force for body heat loss (the difference between core and ambient temperature [13]) and the ease with which heat flows from animal to environment, and that this effect nevertheless predisposes *ob/ob* mice to hypothermia. Defective thermoregulation in *ob/ob* mice, therefore, appears to involve impairment of heat conservation, but not of energy expenditure.

**Fig 6:**
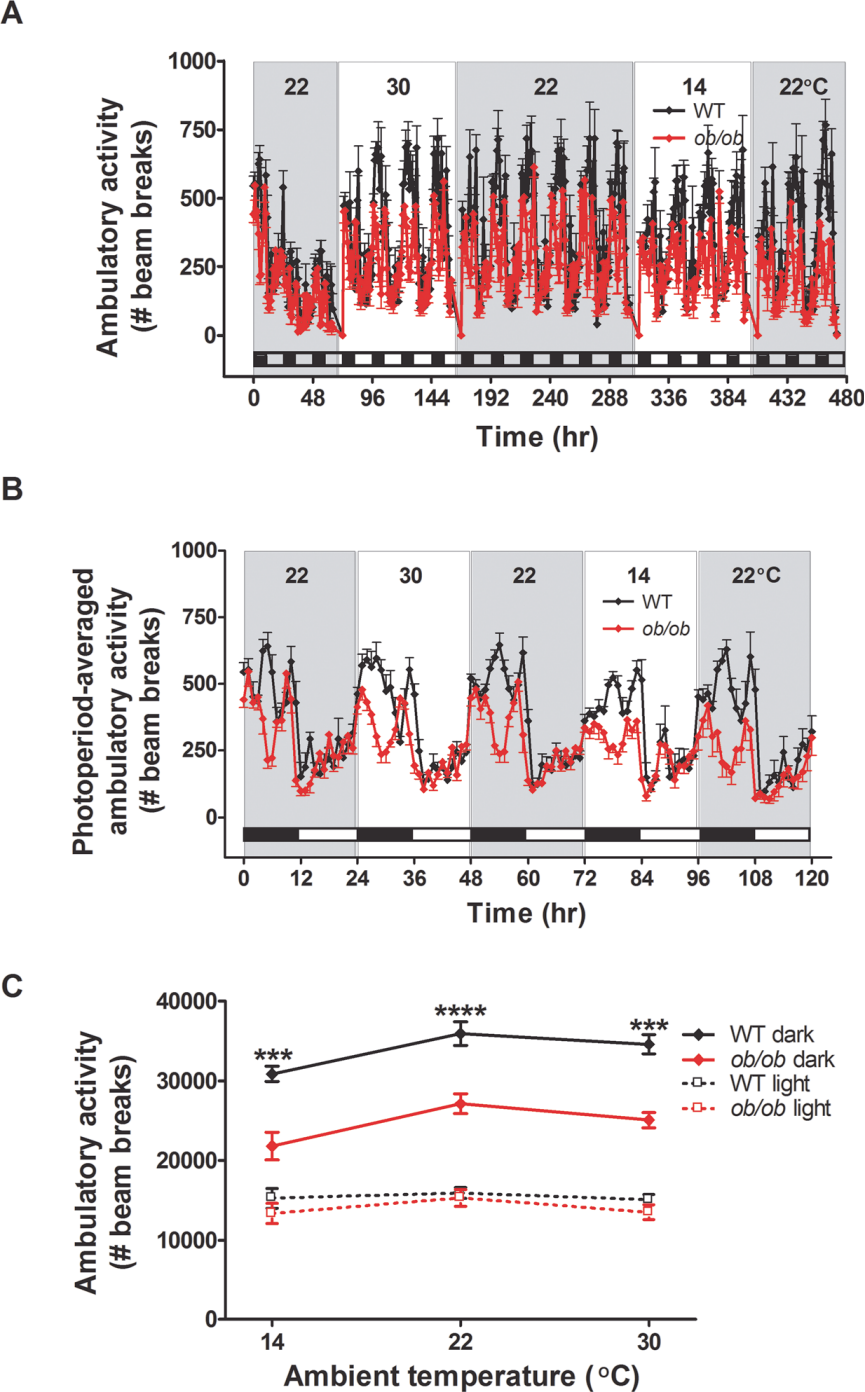
Ambulatory activity levels do not change at different ambient temperatures. (**A**) Ambulatory activity across all photoperiods, (**B**) photoperiod-averaged ambulatory activity and (**C**) the relationship between ambulatory activity and ambient temperature in adult male *ob/ob* mice and wild-type (WT) littermate controls housed under different ambient temperature conditions (n = 8/group). Mean±SEM. ****p<0.0001, ***p<0.001.

Unlike the robust changes of energy expenditure observed in both genotypes, ambulatory activity did not change significantly in response changing thermal environments. As expected, we found a pronounced diurnal rhythm in ambulatory activity in both genotypes, the majority of which occurred during the dark cycle. Furthermore, ambulatory activity was reduced in *ob/ob* mice relative to WT littermates [23] during the dark cycle, but not during the light cycle when ambulatory activity was low in both genotypes. However, ambulatory activity levels did not change in either *ob/ob* or WT animals across the 3 different ambient temperatures (Fig. 6). Changes of physical activity are therefore unlikely to play an important role in the adaptive response to changing environmental temperatures, a conclusion supported by evidence that increments in heat production due to increased movement tend to be offset by elevated convective heat loss [13].

## Discussion

Like other homeothermic animals, mice are endowed with the ability to activate autonomic and behavioral effectors that collectively maintain average body temperature within narrow limits, even in the face of rapid and marked changes of their thermal environment. This ability to preserve euthermia is critically dependent on robust connectivity between thermoregulatory and energy homeostasis systems that permits the thermoregulatory imperative to be fulfilled without fluctuations in body fat stores that can compromise organismal survival. In particular, thermoregulatory demands must be coupled to caloric intake if body weight stability is to be maintained. The current studies were conducted to more fully characterize this coupling process and to investigate the role played by the adipose tissue hormone leptin. Our results extend previous evidence [4,5] that in response to an increase or decrease of ambient temperature, normal mice, but not *ob/ob* mice, maintain their core temperature without significant changes of body lean or fat mass. Among the major new findings gleaned from our studies is that in both WT and *ob/ob* mice, energy expenditure and ambient temperature are strongly and negatively related to one another, and that while a similar negative relationship exists between energy intake and ambient temperature in control mice, this relationship is severely disrupted in *ob/ob* mice. Further, our finding of a pronounced increase of thermal conductance in *ob/ob* mice implies a physiological role for leptin in the control of heat dissipation to the environment. Together, these findings demonstrate *1)* the remarkable capacity of normal mice to match energy intake to energy expenditure (i.e., to display intact energy homeostasis) in response to different thermal conditions, and *2)* that in response to changes of ambient temperature, adaptive changes of food intake and thermal conductance, but not energy expenditure, require intact leptin signaling.

The importance of the coupling of energy homeostasis to thermoregulation has been alluded to previously, including the effect of cold exposure to increase food intake [18,24]. Consistent with this work, we observed that in WT mice, energy intake increases with decreasing environmental temperatures. This hyperphagic response enables mice that are expending a considerable amount of energy on thermoregulatory thermogenesis to supply the energy needed to meet heightened energy needs. Conversely, under thermoneutral conditions when, by definition, core body temperature can be maintained without the need for thermoregulatory thermogenesis, energy intake in normal mice is proportionately reduced. As a consequence, normal animals maintain stable energy balance, body weight and body composition irrespective of whether they are housed in a cool temperature or at thermoneutrality [17,18]. A key, new finding is that the ability to adjust food intake in response to different ambient temperatures appears to require intact leptin signaling. Consequently, *ob/ob* mice rely on catabolism of body fat and lean mass to generate the energy required for heat production at cooler temperatures and as a result, experience pronounced losses of body weight.

Such an effect would surely be deleterious if sustained over the long-term, ultimately giving rise to the distinctive state of reduced energy expenditure, body temperature, and physical activity known as torpor [25]. Indeed, leptin-deficient animals are predisposed to torpor [26], and although previous studies have reported that both leptin-deficient *ob/ob* mice [17] and leptin receptor-deficient Zucker *fa/fa* rats [24] fail to increase their food intake in response to cold exposure, these findings were reported in isolation, in the absence of the comprehensive assessment of energy balance, body composition and body temperature that we provide here. Using this approach, we identified a strong inverse relationship between ambient temperature and energy expenditure that, surprisingly, is largely preserved in *ob/ob* mice and that, whereas a similar relationship between ambient temperature and energy intake exists in normal mice, this relationship is absent in mice lacking leptin. Thus, our work shows for the first time that in response to changes of ambient temperature, adaptive changes of energy intake, but not energy expenditure require leptin signaling.

The failure of *ob/ob* mice to maintain core temperature in response to cold exposure, despite increasing energy expenditure appropriately, is noteworthy [4]. This defect is not secondary to obesity per se [5], nor does it reflect a failure to increase energy intake in response to cold stress, since the hyperphagia characteristic of *ob/ob* mice fails to correct their thermoregulatory defect when housed at 22°C. Instead, we interpret this uncoupling of food intake and thermoregulatory requirements as a direct consequence of leptin deficiency. Similarly, *ob/ob* mice display impaired activation of brown adipose tissue (BAT), a key tissue for non-shivering facultative thermogenesis [1,27], both at room temperature and in response to cold exposure [27,28]. Conversely, exogenous leptin administration increases sympathetic nervous system (SNS) outflow to BAT, which induces expression of uncoupling protein-1 (Ucp1) in BAT to generate heat [29,30].

Despite evidence of a requirement for leptin in SNS-mediated BAT activation, our work therefore reveals that the thermoregulatory failure of *ob/ob* mice is not due to an inability to increase energy expenditure. Rather, the defect appears to involve the failure to translate appropriately increased energy expenditure into effective maintenance of euthermia. This interpretation in turn suggests that leptin is required for cold-induced activation of heat conservation effectors, a major point of thermoregulatory control that in mice involves autonomic regulation of blood flow via the tail artery. In support of this hypothesis, we found that whole body thermal conductance was higher in *ob/ob* mice than WT controls across each of the three different temperatures. Based on an allometric analysis of thermal conductance, we find that leptin deficiency only minimally alters the fundamental mathematical parameter set that dictates the relationship between the driving force for heat loss (core minus ambient temperature) and thermal conductance. This analysis suggests that although thermal conductance is increased in *ob/ob* mice across all ambient temperatures studied, their ability to mount an adaptive change of thermal conductance in response to changes of ambient temperature is fundamentally intact. Additional studies are warranted to identify mechanisms underlying this defect.

These findings collectively support the hypothesis that leptin’s role in thermoregulation is directed more at the retention of than the generation of body heat in the defense of euthermia. Thus, although leptin is required for cold-induced BAT activation [27,28], our findings suggest that *1)* leptin-deficient mice compensate for this defect by increasing energy expenditure via other mechanisms, and *2)* although leptin-independent mechanisms for heat generation also exist, such mechanisms do not exist for heat conservation (e.g., by vasoconstriction) [31]. Although the mechanism(s) underlying increased energy expenditure in cold-exposed *ob/ob* mice remains uncertain, previous evidence suggests that these animals rely on shivering to generate heat [32], analogous to the phenotype of mice lacking uncoupling protein-1 (UCP1), a key mediator of BAT thermogenesis [33–35]. If so, it is also possible that a heightened reliance of *ob/ob* mice on shivering thermogenesis contributes to their inability to increase feeding behavior during cold exposure. Although thermoregulatory behaviors such as shivering or changes in posture (i.e. “balling up”) were not monitored in the current study, future studies are planned to more fully delineate how leptin signaling channels cold-induced increases of energy expenditure towards the maintenance of core temperature.

Besides basal metabolic rate, which reflects energy expended in support of normal metabolism and also produces heat for homeothermy, total energy expenditure includes contributions from both voluntary and involuntary physical activity (including fidgeting, posture, etc…) and energetic cost associated with the absorption, digestion and processing of food known as diet-induced thermogenesis [36]. In this context, we note that ambulatory activity does not appear to contribute substantially to adjustments of energy expenditure induced by changes of ambient temperature in either normal or leptin-deficient mice. Specifically, although ambulatory activity undoubtedly contributes to total energy expenditure [37], we found that reducing the thermoregulatory energy expenditure demands by increasing the ambient temperature failed to lower ambulatory activity, while conversely, mild cold stress did not increase ambulatory activity. Stated differently, physical activity does not appear to be a regulated effector for increasing energy expenditure in the defense of body temperature in mice. Diet-induced thermogenesis is also unlikely to contribute importantly to changes of energy expenditure during cold exposure, since this comprises only a small fraction of total energy expenditure (5–10%). Consequently, even a 50% increase of food intake would be expected to produce only a ∼5% increase of total energy expenditure, whereas total energy expenditure increases by at least 50% in normal mice transitioning from a thermoneutral to a cool (14°C) environment. We therefore view the robust increase of food intake induced by cold exposure in normal mice as more of a compensatory response to meet the needs of increased energy expenditure than as a contributor to increased energy expenditure *per se*.

The hypothesis that reduced energy expenditure contributes to the obese phenotype of *ob/ob* mice is based on evidence that *i) ob/ob* mice become obese even when pair-fed to the intake of lean controls [38], *ii)* leptin treatment induces weight loss in excess of what can be explained by reduced caloric intake [39], *iii)* when measured using indirect calorimetry, energy expenditure increases in both normal mice and *ob/ob* mice in response to leptin administration [40–42], *iv)* when housed at thermoneutrality, *ob/ob* mice still become fatter than normal mice even when their hyperphagia is prevented by pair-feeding [18], suggesting an inherent increase of metabolic efficiency, and *v)* BAT mass and UCP-1 expression are reduced in *ob/ob* relative to normal mice [28] and these effects are reversed by leptin administration [29]. In view of our findings, we suggest that although reduced energy expenditure likely contributes to obesity pathogenesis in leptin-deficient animal models, this defect should not be interpreted to suggest that such animals are incapable of increasing energy expenditure when called upon to protect falling core temperature in cold environments. To the contrary, leptin-deficient mice appear to do this as effectively as normal mice, even if the mechanisms underlying cold-induced increases of heat production are not the same (which remains to be investigated).

Our findings also raise interesting questions regarding the pathogenesis of hyperphagia associated with leptin deficiency. Specifically, our data and others suggest that even when *ob/ob* mice are housed at room temperature, their core temperature is reduced and compensatory responses activated. Thus, hyperphagia in this setting could arise in part as a manifestation of the adaptive response to reduced core body temperature. Since the thermogenic defect is largely corrected when *ob/ob* mice are housed at thermoneutrality (Fig. 1 and [43]), and yet they remain hyperphagic and continue to gain weight in excess of what is observed in controls, we conclude that hyperphagia in *ob/ob* mice is not driven primarily by their reduced core body temperature.

The finding that BAT is activated in response to cold in humans, as it is in rodents [44–48], has stimulated interest in the notion that cold exposure may be a useful way to increase energy expenditure and thereby prevent or treat obesity [49]. Since cold exposure increases food intake, however, our data suggest that this hyperphagic response must be contained for weight loss to be induced by cold exposure and/or BAT activation. Consistent with this concept, modest intermittent cold exposure activates BAT and increases energy expenditure in mice, but fails to change body weight or body composition [50]. Recent work in humans shows that BAT activity and energy expenditure increased following daily, 2-h cold exposure (17°C) for 6 wk, and this effect was associated with reduced body fat mass but was insufficient to induce weight loss [51]. Increased physical activity (e.g., exercise training) has similar effects to reduce fat mass but not body weight in obese humans [52]. Thus, although increasing thermogenesis through any of several means can confer metabolic benefit, improve body composition, and prevent weight gain, it may not be particularly effective for obesity treatment [52].

These comments are also germane to the notion that cold-induced thermogenesis can challenge the “adipostat hypothesis” of body weight control [53]. For example, mice fed a high-fat become obese when housed at a thermoneutral environmental temperature but are protected from diet-induced obesity when housed at a lower ambient temperature [54,55], suggesting that directing extra energy to the maintenance of euthermia prevents positive energy balance and thereby protects against obesity in normal animals. However, this outcome is not evident when animals are consuming chow [54], which taken together with our findings, raises the intriguing possibility that diet-induced obesity derives at least in part from defective coupling of energy homeostasis to thermoregulation. One mechanism with the potential to explain this outcome is that consumption of the high-fat diet induces “leptin resistance” and/or injury and gliosis [56]) affecting hypothalamic neuronal systems that couple energy intake to thermogenic energy requirements. Future studies are warranted to explore this important issue.

In conclusion, we report that the coupling of thermoregulation to energy homeostasis requires intact leptin signaling. We observed a strong negative relationship between energy expenditure and ambient temperature in both normal and *ob/ob* mice, and while a very similar relationship exists between energy intake and ambient temperature in normal mice, this relationship is disrupted in *ob/ob* mice. Based on our finding that hypothermia in *ob/ob* mice is associated with increased thermal conductance, our findings also implicate leptin in the physiological control of mechanisms governing dissipation of body heat to the environment. A greater understanding of the mechanisms that underlie the coupling of thermoregulation to energy homeostasis may lead to more effective treatments for obesity.

## Supporting Information

S1 Fig Allometric models for energy expenditure as functions of the core minus ambient temperature difference. (DOCX)

## References

[1] Silva JE (2006) Thermogenic mechanisms and their hormonal regulation. Physiol Rev 86: 435–464. 16601266 10.1152/physrev.00009.2005

[2] Schwartz MW, Woods SC, Porte D Jr., Seeley RJ, Baskin DG (2000) Central nervous system control of food intake. Nature 404: 661–671. 10766253 10.1038/35007534

[3] Rezai-Zadeh K, Munzberg H (2013) Integration of sensory information via central thermoregulatory leptin targets. Physiol Behav 121: 49–55. 10.1016/j.physbeh.2013.02.014 23458626 PMC3683124

[4] Trayhurn P, Thurlby PL, James WP (1976) A defective response to cold in the obese (obob) mouse and the obese Zucker (fafa) rat [proceedings]. Proc Nutr Soc 35: 133A 1036201

[5] Trayhurn P, Thurlby PL, James WP (1977) Thermogenic defect in pre-obese ob/ob mice. Nature 266: 60–62. 840297 10.1038/266060a0

[6] Taicher GZ, Tinsley FC, Reiderman A, Heiman ML (2003) Quantitative magnetic resonance (QMR) method for bone and whole-body-composition analysis. Anal Bioanal Chem 377: 990–1002. 13680051 10.1007/s00216-003-2224-3

[7] Kaiyala KJ, Morton GJ, Thaler JP, Meek TH, Tylee T, Ogimoto K, et al (2012) Acutely decreased thermoregulatory energy expenditure or decreased activity energy expenditure both acutely reduce food intake in mice. PLoS One 7: e41473 10.1371/journal.pone.0041473 22936977 PMC3425585

[8] Morton GJ, Kaiyala KJ, Fisher JD, Ogimoto K, Schwartz MW, Wisse BE (2011) Identification of a physiological role for leptin in the regulation of ambulatory activity and wheel running in mice. Am J Physiol Endocrinol Metab 300: E392–401. 10.1152/ajpendo.00546.2010 21062956 PMC3043625

[9] Morton GJ, Thatcher BS, Reidelberger RD, Ogimoto K, Wolden-Hanson T, Baskin DG, et al (2012) Peripheral oxytocin suppresses food intake and causes weight loss in diet-induced obese rats. Am J Physiol Endocrinol Metab 302: E134–144. 10.1152/ajpendo.00296.2011 22008455 PMC3328087

[10] Weir JB (1949) New methods for calculating metabolic rate with special reference to protein metabolism. J Physiol 109: 1–9. 15394301 10.1113/jphysiol.1949.sp004363 PMC1392602

[11] Kaiyala KJ, Morton GJ, Leroux BG, Ogimoto K, Wisse B, Schwartz MW (2010) Identification of body fat mass as a major determinant of metabolic rate in mice. Diabetes 59: 1657–1666. 10.2337/db09-1582 20413511 PMC2889765

[12] Kaiyala KJ, Schwartz MW (2010) Toward a more complete (and less controversial) understanding of energy expenditure and its role in obesity pathogenesis. Diabetes 60: 17–23. 10.2337/db10-0909 PMC3012169 21193735

[13] Gordon CJ (1993) Temperature regulation in laboratory rodents New York: Cambridge University Press.

[14] Kaiyala KJ (2014) Mathematical model for the contribution of individual organs to non-zero y-intercepts in single and multi-compartment linear models of whole-body energy expenditure. PLoS One 9: e103301 10.1371/journal.pone.0103301 25068692 PMC4113365

[15] West GB, Brown JH, Enquist BJ (1997) A general model for the origin of allometric scaling laws in biology. Science 276: 122–126. 9082983 10.1126/science.276.5309.122

[16] Romsos DR, Ferguson D, Vander Tuig JG (1985) Effects of a warm environment on energy balance in obese (ob/ob) mice. Metabolism 34: 931–937. 4046837 10.1016/0026-0495(85)90141-6

[17] Smith CK, Romsos DR (1984) Cold acclimation of obese (ob/ob) mice: effects of energy balance. Metabolism 33: 853–857. 6472117 10.1016/0026-0495(84)90114-8

[18] Thurlby PL, Trayhurn P (1979) The role of thermoregulatory thermogenesis in the development of obesity in genetically-obese (ob/ob) mice pair-fed with lean siblings. Br J Nutr 42: 377–385. 508700 10.1079/bjn19790127

[19] Refinetti R, Menaker M (1992) The circadian rhythm of body temperature. Physiol Behav 51: 613–637. 1523238 10.1016/0031-9384(92)90188-8

[20] Refinetti R (2010) The circadian rhythm of body temperature. Front Biosci (Landmark Ed) 15: 564–594. 20036834 10.2741/3634

[21] McNab BK (1980) On estimating thermal conductance in endotherms. Physiol Zool 53: 145–156.

[22] Schleucher E, Withers PC (2001) Re-evaluation of the allometry of wet thermal conductance for birds. Comp Biochem Physiol A Mol Integr Physiol 129: 821–827. 11440868 10.1016/s1095-6433(01)00356-7

[23] Dauncey MJ (1986) Activity-induced thermogenesis in lean and genetically obese (ob/ob) mice. Experientia 42: 547–549. 3709761 10.1007/BF01946696

[24] Armitage G, Harris RB, Hervey GR, Tobin G (1984) The relationship between energy expenditure and environmental temperature in congenitally obese and non-obese Zucker rats. J Physiol 350: 197–207. 6747849 10.1113/jphysiol.1984.sp015196 PMC1199264

[25] Melvin RG, Andrews MT (2009) Torpor induction in mammals: recent discoveries fueling new ideas. Trends Endocrinol Metab 20: 490–498. 10.1016/j.tem.2009.09.005 19864159 PMC2788021

[26] Himms-Hagen J (1985) Food restriction increases torpor and improves brown adipose tissue thermogenesis in ob/ob mice. Am J Physiol 248: E531–539. 4039535 10.1152/ajpendo.1985.248.5.E531

[27] Hogan S, Himms-Hagen J (1980) Abnormal brown adipose tissue in obese (ob/ob) mice: response to acclimation to cold. Am J Physiol 239: E301–E309. 7425122 10.1152/ajpendo.1980.239.4.E301

[28] Himms-Hagen J (1985) Defective brown adipose tissue thermogenesis in obese mice. Int J Obes 9 Suppl 2: 17–24. 4066136

[29] Scarpace PJ, Matheny M (1998) Leptin induction of UCP1 gene expression is dependent on sympathetic innervation. Am J Physiol 275: E259–264. 9688627 10.1152/ajpendo.1998.275.2.E259

[30] Haynes WG, Morgan DA, Walsh SA, Mark AL, Sivitz WI (1997) Receptor-mediated regional sympathetic nerve activation by leptin. J Clin Invest 100: 270–278. 9218503 10.1172/JCI119532 PMC508189

[31] Vinter J, Hull D, Batt RA, Tyler DD (1988) The effect of limit feeding on thermogenesis and thermoregulation in genetically obese (ob/ob) mice during cold exposure. Int J Obes 12: 111–117. 3384557

[32] Jansky L, Bartunkova R, Zeisberger E (1967) Acclimation of the white rat to cold: noradrenaline thermogenesis. Physiol Bohemoslov 16: 366–372. 4228115

[33] Enerback S, Jacobsson A, Simpson EM, Guerra C, Yamashita H, Harper ME, et al (1997) Mice lacking mitochondrial uncoupling protein are cold-sensitive but not obese. Nature 387: 90–94. 9139827 10.1038/387090a0

[34] Golozoubova V, Cannon B, Nedergaard J (2006) UCP1 is essential for adaptive adrenergic nonshivering thermogenesis. Am J Physiol Endocrinol Metab 291: E350–357. 16595854 10.1152/ajpendo.00387.2005

[35] Golozoubova V, Hohtola E, Matthias A, Jacobsson A, Cannon B, Nedergaard J (2001) Only UCP1 can mediate adaptive nonshivering thermogenesis in the cold. Faseb J 15: 2048–2050. 11511509 10.1096/fj.00-0536fje

[36] Rosenbaum M, Leibel RL (2010) Adaptive thermogenesis in humans. Int J Obes (Lond) 34 Suppl 1: S47–55. 20935667 10.1038/ijo.2010.184 PMC3673773

[37] Morrison SD (1968) The constancy of the energy expended by rats on spontaneous activity, and the distribution of activity between feeding and non-feeding. J Physiol 197: 305–323. 5716848 10.1113/jphysiol.1968.sp008561 PMC1351801

[38] Chlouverakis C (1970) Induction of obesity in obese-hyperglycaemic mice on normal food intake. Experientia 26: 1262–1263. 5274345 10.1007/BF01898005

[39] Levin N, Nelson C, Gurney A, Vandlen R, de Sauvage F (1996) Decreased food intake does not completely account for adiposity reduction after ob protein infusion. Proc Natl Acad Sci U S A 93: 1726–1730. 8643697 10.1073/pnas.93.4.1726 PMC40010

[40] Campfield LA, Smith FJ, Guisez Y, Devos R, Burn P (1995) Recombinant mouse OB protein: Evidence for a peripheral signal linking adiposity and central neural networks. Science 269: 546–549. 7624778 10.1126/science.7624778

[41] Halaas JL, Gajiwala KS, Maffei M, Cohen SL, Chait BT, Rabinowitz D, et al (1995) Weight-reducing effects of the plasma protein encoded by the *obese* gene. Science 269: 543–546. 7624777 10.1126/science.7624777

[42] Pelleymounter MA, Cullen MJ, Baker MB, Hecht R, Winters D, Boone T, et al (1995) Effects of the *obese* gene product on body weight regulation in *ob/ob* mice. Science 269: 540–543. 7624776 10.1126/science.7624776

[43] Trayhurn P, James WP (1978) Thermoregulation and non-shivering thermogenesis in the genetically obese (ob/ob) mouse. Pflugers Arch 373: 189–193. 565045 10.1007/BF00584859

[44] Cypess AM, Lehman S, Williams G, Tal I, Rodman D, Goldfine AB, et al (2009) Identification and importance of brown adipose tissue in adult humans. N Engl J Med 360: 1509–1517. 10.1056/NEJMoa0810780 19357406 PMC2859951

[45] Saito M, Okamatsu-Ogura Y, Matsushita M, Watanabe K, Yoneshiro T, Nio-Kobayashi J, et al (2009) High incidence of metabolically active brown adipose tissue in healthy adult humans: effects of cold exposure and adiposity. Diabetes 58: 1526–1531. 10.2337/db09-0530 19401428 PMC2699872

[46] van der Lans AA, Hoeks J, Brans B, Vijgen GH, Visser MG, Vosselman MJ, et al (2013) Cold acclimation recruits human brown fat and increases nonshivering thermogenesis. J Clin Invest 123: 3395–3403. 10.1172/JCI68993 23867626 PMC3726172

[47] van Marken Lichtenbelt WD, Vanhommerig JW, Smulders NM, Drossaerts JM, Kemerink GJ, Bouvy ND, et al (2009) Cold-activated brown adipose tissue in healthy men. N Engl J Med 360: 1500–1508. 10.1056/NEJMoa0808718 19357405

[48] Virtanen KA, Lidell ME, Orava J, Heglind M, Westergren R, Niemi T, et al (2009) Functional brown adipose tissue in healthy adults. N Engl J Med 360: 1518–1525. 10.1056/NEJMoa0808949 19357407

[49] Lichtenbelt W, Kingma B, van der Lans A, Schellen L (2014) Cold exposure—an approach to increasing energy expenditure in humans. Trends Endocrinol Metab 25: 165–167. 10.1016/j.tem.2014.01.001 24462079

[50] Ravussin Y, Xiao C, Gavrilova O, Reitman ML (2014) Effect of intermittent cold exposure on brown fat activation, obesity, and energy homeostasis in mice. PLoS One 9: e85876 10.1371/journal.pone.0085876 24465761 PMC3895006

[51] Yoneshiro T, Aita S, Matsushita M, Kayahara T, Kameya T, Kawai Y, et al (2013) Recruited brown adipose tissue as an antiobesity agent in humans. J Clin Invest 123: 3404–3408. 10.1172/JCI67803 23867622 PMC3726164

[52] Thorogood A, Mottillo S, Shimony A, Filion KB, Joseph L, Genest J, et al (2011) Isolated aerobic exercise and weight loss: a systematic review and meta-analysis of randomized controlled trials. Am J Med 124: 747–755. 10.1016/j.amjmed.2011.02.037 21787904

[53] Cannon B, Nedergaard J (2009) Thermogenesis challenges the adipostat hypothesis for body-weight control. Proc Nutr Soc 68: 401–407. 10.1017/S0029665109990255 19775494

[54] Rippe C, Berger K, Boiers C, Ricquier D, Erlanson-Albertsson C (2000) Effect of high-fat diet, surrounding temperature, and enterostatin on uncoupling protein gene expression. Am J Physiol Endocrinol Metab 279: E293–300. 10913028 10.1152/ajpendo.2000.279.2.E293

[55] Ravussin Y, LeDuc CA, Watanabe K, Leibel RL (2012) Effects of ambient temperature on adaptive thermogenesis during maintenance of reduced body weight in mice. Am J Physiol Regul Integr Comp Physiol 303: R438–448. 10.1152/ajpregu.00092.2012 22761182 PMC3423991

[56] Thaler JP, Yi CX, Schur EA, Guyenet SJ, Hwang BH, Dietrich MO, et al (2012) Obesity is associated with hypothalamic injury in rodents and humans. J Clin Invest 122: 153–162. 10.1172/JCI59660 22201683 PMC3248304

