# Supplementary material for: Leptin Signaling Is Required for Adaptive Changes in Food Intake, but Not Energy Expenditure, in Response to Different Thermal Conditions
Source: PLoS One. 2015 Mar 10;10(3):e0119391. doi: 10.1371/journal.pone.0119391 (PMC4355297; doi:10.1371/journal.pone.0119391)
Supplement: S1 Fig — (DOCX) [file pone.0119391.s001.docx]

**Online Supplement for “*Leptin signaling is required for adaptive changes in food intake, but not energy expenditure, in response to different thermal conditions”* by Karl J. Kaiyala, Kayoko Ogimoto, Jarrell T. Nelson, Michael W. Schwartz and Gregory J. Morton.**

**Appendix S1.**

**Allometric models for energy expenditure as functions of the core minus ambient temperature difference.**

Regression of mean 24 h energy expenditure (EE) values on the mean difference between core temperature and ambient temperature (*Tc-Ta*) revealed *1)* a steeper slope in *ob/ob* than WT mice and *2)* that the mean square error of the regression residuals increased in proportion to the temperature difference, as would be expected if the underlying mathematical relationship conformed to a classical multiplicative power (allometric) form. Given the ubiquity of allometric scaling in biophysical processes [14, 15], we sought to examine the relationship between the natural log of energy expenditure (EE) and the natural log of (*Tc-Ta*) (Figure 4D). This analysis showed that the regression slopes between the two groups are almost identical and that the residual error is both similar between groups and is essentially constant across temperature difference values (see Figure 4D). Accordingly, we fit an analysis of covariance model to the log-log data (Figure 4D) and exponentiated the parameter estimates to obtain allometric models [15] for the dependence of energy expenditure on (*Tc-Ta*).

The parameter estimates (± SEM) of the log-log equation (when EE is in kcal/h and temperature in oC) are:

ln(EE)=(-2.204±0.0922) + (0.462±0.0927) x ln (*Tc-Ta*) + (0.275±0.0763) for *ob/ob*; 0 for WT. (The term for group is significant at p=0.004 and the coefficient for ln(Tc-Ta) is significant at p<0.0005).

Accordingly, the allometric models for EE as functions of (*Tc-Ta*) are:

WT: EE = 0.110 x (*Tc-Ta*)^0.46^

*ob/ob*: EE = 0.145 x (*Tc-Ta*)^0.46^

These equations, which are illustrated in Figure S1, show that EE is 31.8% higher in *ob/ob* compared to WT mice at any particular value of (*Tc-Ta*).

Differentiating the energy expenditure equations with respect to (*Tc-Ta*) we get:

WT:  *dEE*  = 0.110 x 0.46 x (*Tc-Ta*)^-0.54^

*d(Tc-Ta)*

*ob/ob*:  *dEE*  = 0.110 x 0.46 x (*Tc-Ta*)^-0.54^

*d(Tc-Ta)*

This analysis again indicates that the increase in EE per unit increase in (*Tc-Ta*) is 31.8% higher in *ob/ob* compared to WT mice at any particular value of (*Tc-Ta*).

Conductance (K) reflects the ease with which flows down a temperature gradient and is defined as energy expenditure per degree of (*Tc-Ta*), calculated by dividing both sides of the energy expenditure equation by (*Tc-Ta*)^-1^. This yields the following allometric models for conductance:

WT: K = 0.110 x (*Tc-Ta*)^-0.54^

*ob/ob*: K = 0.145 x (*Tc-Ta*) ^-0.54^

and indicates that conductance is 31.8% higher in *ob/ob* compared to WT mice at any particular value of (*Tc-Ta*).

To summarize, 24h EE, the rate of change of 24h EE, and whole body 24h thermal conductance are each estimated to be 31.8% higher in *ob/ob* compared to WT mice across all values of (*Tc-Ta*) included in our study.

Conductance has been modeled in terms of allometric functions of body mass [22] and as linear functions of ambient temperature [21] but we are unaware of previous models involving the core minus ambient temperature difference as the predictor variable.

**
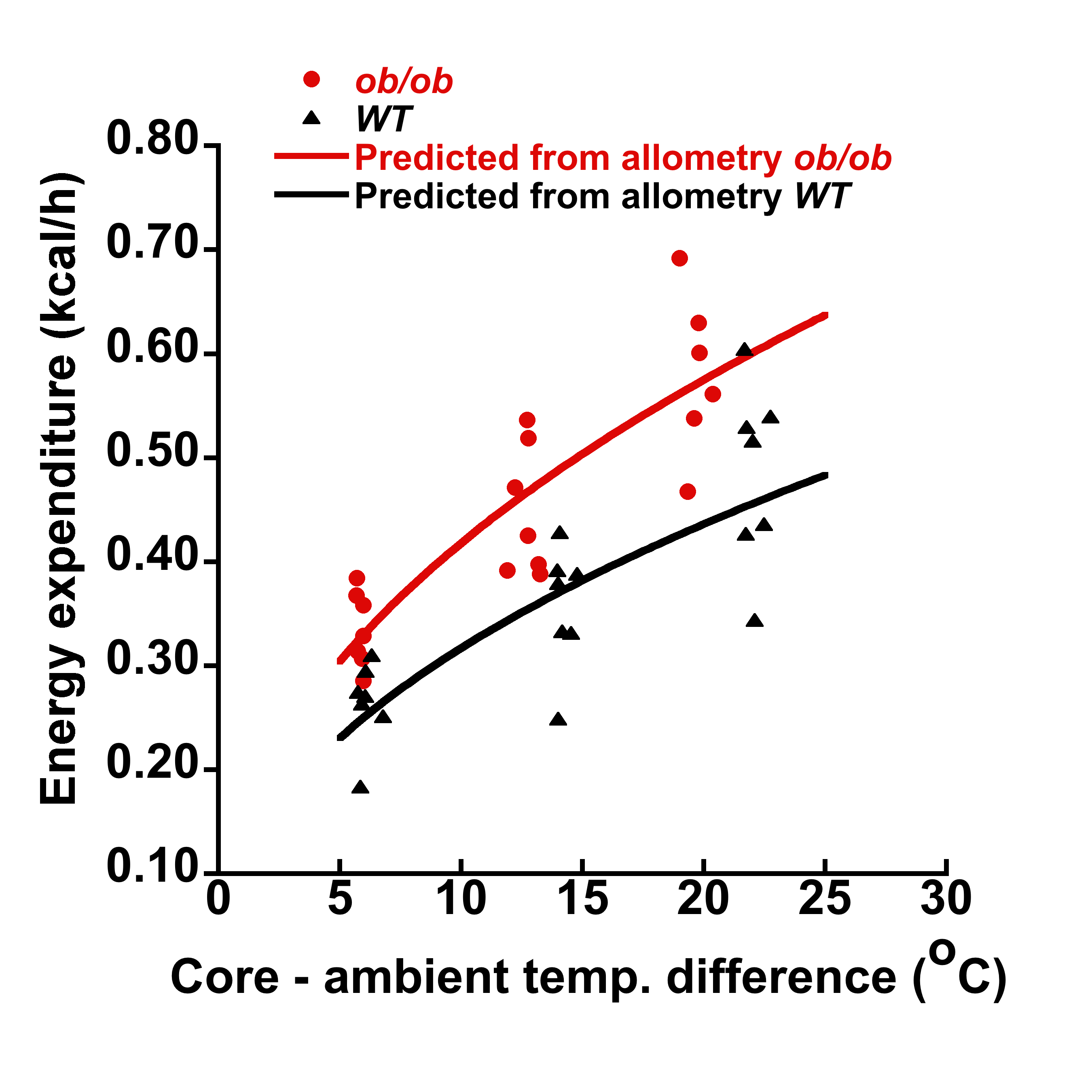
**

**Figure S1. Energy expenditure and allometric curve fits for *ob/ob* and WT mice vs. the core minus ambient temperature difference.**
